# Understanding Mantle Edge Pigmentation Through Comprehensive Transcriptomic Profiling of the Chilean Oyster (*Ostrea chilensis*)

**DOI:** 10.3390/biology14020145

**Published:** 2025-01-30

**Authors:** Camila Godoy-Diaz, Katalina Llanos-Azócar, Gonzalo J. Ruiz-Tagle, Jorge E. Toro, Pablo A. Oyarzún, Juan A. Valdés

**Affiliations:** 1 Departamento de Ciencias Biológicas, Facultad de Ciencias de la Vida, Universidad Andres Bello, Santiago 8370146, Chile; 2 Interdisciplinary Center for Aquaculture Research (INCAR), Concepción 4030000, Chile; 3 Centro de Investigación Marina Quintay (CIMARQ), Universidad Andres Bello, Valparaíso 2340000, Chile; 4 Instituto de Ciencias Marinas y Limnológicas (ICML), Universidad Austral de Chile, Independencia 631, Valdivia 5090000, Chile

**Keywords:** ABC transporters, cAMP/Notch/Calcium signaling pathway, endocytosis, flat oyster, mantle edge pigmentation, RNA-seq, tyrosine/tryptophan metabolism

## Abstract

The Chilean oyster (*Ostrea chilensis*) is a flat oyster native to Chile and New Zealand. Over-exploitation has led to local extinctions in some areas. Two phenotypes, distinguished by dark or white mantle edge pigmentation, have been identified, with the dark-edged mantle oysters being more commercially valuable due to perceived quality. In this study, transcriptomic data were obtained from the mantles of both phenotypes. Total RNA was extracted of adult Chilean Oyster mantle, and samples were sequenced using HiSeq X Illumina technology. A total of 935,620,583 paired-end reads were de novo assembled, 50,908 transcripts produced, and a reference transcriptome created with 37.92% GC content and an N50 of 1929 bp. Functional annotation showed a total of 51,315 GO terms, with 21,322 annotations on Biological Process, 14,578 annotations on Molecular Functions, and 15,415 annotations on Cellular Component. The RNA-seq analysis revealed 746 differentially expressed transcripts, 573 up-regulated and 173 transcripts down-regulated, between dark- and white-mantle edge Chilean Oyster. KEGG enrichment analysis revealed notable differences in the expression profiles allowing the detection of differential expressed transcripts associated with specific pathways such as Ribosome, Citrate cycle, and Protein processing in endoplasmic reticulum. Other interesting differentially represented pathways include Tyrosine metabolism, Tryptophan metabolism, cAMP signaling pathway, ABC transporters, Notch signaling pathway, Endocytosis, and Calcium signaling pathway. This dataset provides a valuable molecular resource for *O. chilensis* and the understanding of the molecular mechanisms involved in mantle edge pigmentation.

## Simple Summary

The Chilean oyster (*Ostrea chilensis*), a species native to Chile and New Zealand, is a valuable resource in aquaculture but faces population declines due to over-exploitation. This study investigates the molecular basis of two distinct phenotypes, characterized by dark or white mantle edge pigmentation, focusing on traits that could influence breeding strategies and stock management in aquaculture. Through the RNA sequencing of mantle tissues, we generated a comprehensive transcriptome, identifying over 50,000 transcripts. Differential gene expression analysis revealed 746 genes with varied activity between the two phenotypes. Pathways associated with pigmentation, metabolism, and cellular signaling, such as the tyrosine and tryptophan metabolism pathways, ribosome function, and calcium signaling, were significantly impacted. The findings enhance the understanding of the genetic and molecular processes underlying phenotypic variation in *O. chilensis*, providing critical data to improve selective breeding programs.

## 1. Introduction

*Ostrea chilensis* (Küster, 1844), known as the Chilean flat oyster, is a bivalve mollusk native to Chile and New Zealand, where it is farmed for commercial purposes [1,2,3]. It is characterized by its slow growth rate, brooding behavior, and limited dispersal potential [4,5,6,7]. In Chile, the farming of this species has prospered mainly in areas near the Island of Chiloé, including localities such as Ancud, Yaldad, Castro, and Calbuco, for approximately 80 years [4,5]. The Chilean oyster is in high demand due to its excellent taste. It is currently an important aquaculture resource with an annual production of 600 tons [8]. Part of this production is sold in the domestic market, while another portion is exported internationally, where the price per ton has exceeded USD 15,000. Exports go to countries such as Costa Rica, Uruguay, and China [9]. However, in the last two decades, the production of Chilean oysters has experienced notable fluctuations, mainly due to overexploitation, leading to local population extinctions and a significant decrease in the size of natural beds [5,10]. This species lives in medium–high environments of coastal and estuarine areas of southern Chile, in shallow waters, typically from low tide to depths of up to 11 m, attached to hard rocky or muddy bottoms in enclosed bays or areas protected from strong waves [4,11].

The Chilean oyster is distinguished by having one flat valve (left) and one concave valve (right). Its shell is typically greenish in color and can grow to over 10 cm in size. Two phenotypes associated with the coloration of the mantle edge have been described, with those having a dark edge achieving the highest commercial value [12]. In the oyster market, dark mantle edge (DME) oysters are associated with pleasant organoleptic characteristics over white mantle edge (WME) oysters. Black-edge oysters cannot be distinguished morphologically at any stage of their life. It is only possible to determine the color of the mantle edge once the oysters are open. In addition, the pigmentation of the mantle edge appears after 15–16 months of age, which makes it difficult to identify this phenotype in the early stages of life [5,6]. This trait is not exclusive to Chilean oysters. For example, in Korea, the Pacific oyster with a black mantle edge is favored by consumers and is sold at a 20% higher price [13], making it a commercially significant trait about which we know little in molecular terms [14]. *Ostrea chilensis* is a protandric hermaphroditic bivalve, maturing first as a male and then changing sex during its life [15,16]. The reproduction of this species involves the release of male gametes into the water, while female gametes or eggs are released into the mantle cavity of the female [17,18]. Once internal fertilization has occurred, egg incubation can last up to approximately seven weeks, with variations attributed to the prevailing environmental conditions during the reproductive season [19]. Although there is much information regarding the biology and reproductive cycle of this species, few studies have been developed on a molecular basis due to the absence of genomic information in databases. Some advances have been made in characterizing the genetic structure and diversity of *O. chilensis* populations using Random Amplified Polymorphic DNA (RAPD) analyses [20], and mitochondrial (Cytb) and nuclear (ITS1) DNA sequence variation [5,21]. A recent molecular study evaluated the proximal biochemical composition and fatty acid profile of the Chilean oyster, revealing a rich composition of omega-3 long-chain polyunsaturated fatty acids [22]. However, the lack of available sequences in databases limits the study of this species.

Over the last decade, high-throughput sequencing technologies have dramatically transformed biological research, enabling the sequencing of DNA and RNA in any organism [23]. RNA-seq has provided comprehensive data on gene expression levels in bivalves under different conditions, helping identify genes involved in growth, reproduction, and pathogen responses [24]. RNA-seq analysis in the Pacific oyster (*Crassostrea gigas*) has improved our understanding of immune responses to *Vibrio alginolyticus* infection and disease resistance [25]. In the same species, comparative studies have elucidated the molecular basis of fast growth, gene expression, alternative splicing, and molecular evolution [26]. Recent research has advanced our understanding of the molecular mechanisms underlying oyster mantle pigmentation. RNA-seq analyses of the Pacific oyster mantle have revealed differential gene expressions linked to pathways such as tyrosine metabolism, melanogenesis, cytochrome P450 activity, endocytosis, and cAMP signaling, all of which are associated with mantle and shell pigmentation [27,28]. This technology has also been used to obtain genomic information on less-studied oyster species, such as the Qatari pearl oyster (*Pinctada imbricata radiata*) [29] and the Iwagaki oyster (*Crassostrea nippona* synonymy *Magallana nippona*) [30]. The objective of this study was to generate the first de novo transcriptome assembly for the mantle of *O. chilensis*, providing useful information for future studies in the aquaculture of this species. Even more, we evaluated the molecular mechanisms associated with phenotypes dark mantle edge (DME) and white mantle edge (WME) mantle pigmentation, revealing interesting details regarding processes associated with Tyrosine and Tryptophan metabolisms, cAMP/Notch/Calcium signaling pathway, ABC transporters, and Endocytosis.

## 2. Materials and Methods

### 2.1. Oysters Maintenance and Experimental Design

The study adhered to animal welfare procedures and was approved by the bioethical committees of the Universidad Andres Bello and the National Agency for Research and Development (ANID) of the Chilean government. Two hundred Chilean oysters with an average weight of 28 ± 4 g and length of 4.10 ± 1.23 cm were collected from Estuario de Quempillén, Chiloe (41°52′ S 73°46′ W, Región de Los Lagos, Chile) and transported to the Centro de Investigación Marina de Quintay (CIMARQ) (33°13′S 71°38′ W, Región de Valparaíso, Chile). Oysters were maintained under natural seawater temperature and pH (12 °C ± 1 °C and pH of 7.9). After a week of acclimation, oysters were randomly divided among three different tanks, which are biological replicates of the Chilean oyster group with 40 oysters per tank. A total of 18 Chilean oysters were dissected; 3 white-edge and 3 dark-edge were selected from each tank, and mantles were collected in RNA later and stored at −80 °C.

### 2.2. Library Construction and RNA Sequencing

Total RNA was obtained from the oyster edge mantle (1 g) using Trizol^®^ (Invitrogen, Carlsbad, CA, USA) following manufacturer recommendations. The total RNA was measured by a Qubit fluorometer using the Qubit RNA BR assay kit (Invitrogen) and RNA integrity was confirmed by the capillary electrophoresis Fragment Analyzer Automated CE System (Advanced Analytical Technologies, Ames, IA, USA). Three cDNA libraries of WE oyster and three cDNA libraries of DE oyster were made using the TruSeq RNA Sample Preparation kit v2 (Illumina^®^, San Diego, CA, USA) following the manufacturer’s recommendations. The library was quantified using the Kapa Library Quantification kit (Roche, NJ, USA) on an AriaMx real-time PCR (qPCR) thermocycler (Agilent, Santa Clara, CA, USA). Libraries were sequenced (2 × 150 bp) using the HiSeq X (Illumina) sequencing platform of Macrogen (Seoul, Korea). The raw data were deposited into the Sequence Read Archive (SRA) available on the NCBI database (SRR30335150; SRR30335149).

### 2.3. Transcriptome De Novo Assembly and Annotation

Raw sequencing reads were trimmed by removing low-quality reads (Q < 30) and sequences with lengths less than 30 bp. De novo assembly was conducted using the CLC Genomics Workbench v23.0.3 (CLC Qiagen, Germantown, MD, USA) using de novo assembly tool. The assembly parameters include a minimum contig size of 500 bp. The % GC and N50 statistics were determined for de novo assembly. To identify Open Reading Frames (ORFs) in the assembly, we run TranSuite software v0.2.3 [31], and to reduce contig redundancy, we run CD-Hit-EST software v4.8.1 [32]. Completeness of the de novo assembly was evaluated with Benchmarking Universal Single-Copy Orthologs (BUSCO) software v5.4.4, using the Metazoa database as per reference [33]. De novo assembly functional annotation was performed against the NCBI Nr database (downloaded October 2023). The annotation was generated with the BLASTx algorithm on the local BLAST server with a threshold of E-value 10^−3^, with matrix BLOSUM62. Functional annotation to obtain Gene Ontology (GO) analysis for transcripts was performed with Blast2GO software (https://www.blast2go.com/) [34].

### 2.4. Differential Expression and GO Enrichment Analyses

To identify differentially expressed transcripts (DETs), reads were mapped to the Chilean oyster assembled transcriptome, using CLC Genomics Workbench, v23.0.3 (CLC Qiagen, Germantown, MD, USA),with the following parameters: mismatches = 2; minimum fraction length = 0.9; minimum fraction similarity = 0.8, and maximum hits per read = 5. Gene expressions were based on reads per kilobase of exon model per million mapped read (RPKM) values. The DEG analytical tool of the CLC Genomic Workbench, v23.0.3 was then used to perform statistical analysis, using the option of “Differential expression in two groups”, “whole transcriptome RNA-seq”, and “TMM normalization method”. Transcripts with absolute fold-change values > 2.0 and FDR-corrected *p*-values < 0.05 were included in the GO and KEGG enrichment analyses. Enrichment analysis and KEGG annotation were performed on the list of differentially expressed transcripts and GO terms to determine the overrepresented processes, considering up and down-expressed transcripts. The differentially regulated genes were categorized based on GO terms for biological processes, molecular functions, and cellular components using the DAVID database [35]. Additionally, the Kyoto Encyclopedia of Genes and Genomes (KEGG) metabolic pathway database was used to build the represented pathways through the KEGG Automatic Annotation Server (KAAS) using the KEGG Orthology (KO) identifiers of the differentially expressed list. To determine a relationship between the DAVID background and *O. Chilensis* DETs, a search in BLASTx was performed against *Crassostrea gigas* Ensembl proteins for major matches with the *O. Chilensis* transcriptome. Ensembl Gene IDs of *C. gigas* were acquired from the resultant Ensembl entries. Custom ID sets were selected for DAVID analysis as the “Background” Standard settings for ease (0.1) and gene count (2).

### 2.5. RNA-Seq Validation by Real-Time PCR

The total RNA extracted was described above. The RNA was quantified using NanoDrop technology (BioTek Instruments, Winooski, VT, USA), selecting the samples with an A260/280 ratio between 1.9 and 2.1. For cDNA synthesis, the residual genomic DNA was removed using RNase-Free DNase (Promega, Madison, WI, USA). Then, RNA was reverse transcribed into cDNA for 60 min at 42 °C using the ImProm-II Reverse Transcription System (Promega). The real-time PCR (qPCR) was performed in an MX3000P thermocycler (Agilent Technologies, Santa Clara, CA, USA) following the MIQE guidelines [36]. Each qPCR reaction mixture contained 7.5 μL of 2 × Brilliant^®^ II SYBR^®^ master mix (Agilent Technologies), 0.75 μL of each primer (250 nM), and 6 μL of cDNA (40-fold diluted) in 15 µL of final volume. All information about primers used in this study is listed in Table S1. The qPCR protocol was realized in triplicate: initial denaturation at 95 °C for 10 min, and 40 cycles of denaturation for 30 s at 95 °C, 30 s of annealing, and 30 s of elongation at 72 °C. To confirm a single PCR product, a melting curve was also performed. The resulting data were expressed in arbitrary units (AU), and analyzed using Q-Gene software (https://www.qgene.org) [37] using 40S ribosomal protein S30 (*fau*) and beta-actin (*actb*) as reference genes.

### 2.6. Statistical Analysis

Significant differences in gene expression between groups were determined by one-way ANOVA followed by Tukey’s test (multiple comparisons). Prior to the analyses, the assumptions of homoscedasticity and normality of the data were tested using the Levene and Kolmogorov–Smirnov tests, respectively. Correlations between RNA-seq and qPCR data were assessed through multiple linear regressions, using coefficients of determination (R2) and *p*-values. All statistical analyses were performed using GraphPad Prism v.5.00 (GraphPad Software, San Diego, CA, USA). ChatGPT (https://chat.openai.com/) was used to edit English orthography, grammar, and redaction in the manuscripts.

## 3. Results

### 3.1. De Novo Assembly and Annotation of Ostrea Chilensis Transcriptome

**Figure 1:**
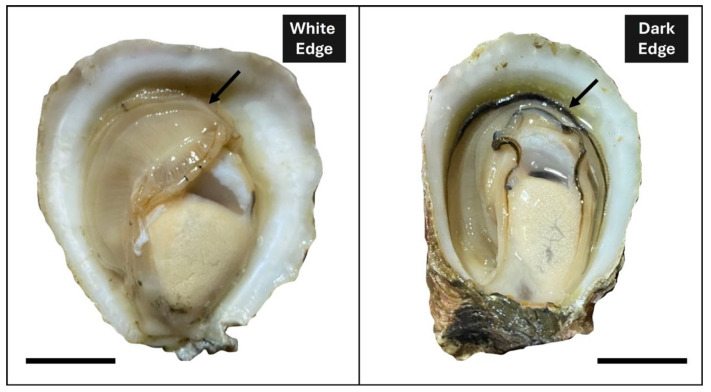
White mantle edge (WME) and dark mantle edge (DME) Chilean oyster (*O. chilensis*) phenotypes.

To generate the first transcriptome available of *O. chilensis*, we used RNA sequencing of mantle edges obtained from three dark (DME) and three white (WME) Chilean oysters (Figure 1).

**Figure 2:**
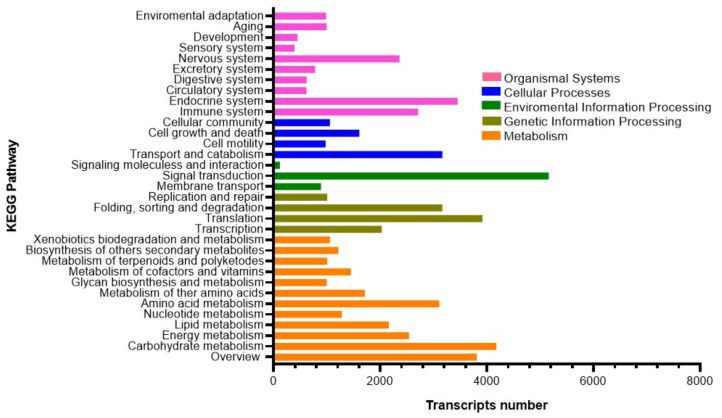
KEGG assignment of transcripts in the mantle transcriptome of *O. chilensis* in the following categories: cellular processes, environmental information processing, genetic information processing, metabolism, and organismal systems.

After quality evaluation and filtering we obtained a total of 935,620,583 pair-end reads, with an average of 155,936,764 pair-end reads per library, that were used for de novo transcriptome assembly (Table S2). The raw data are available at NCBI under BioProject code PRJNA1150665. The de novo assembly generated a total of 50,908 transcripts, with 37.92% of GC content and an N50 value of 1,929 (Table S3). The self-mapping rate for the final assembly presented a 94.31% mapping, which represents a reliable value for a de novo assembly. In terms of ORF detection, we determined that 83,611 ORFs were present in the assembly. To analyze the completeness in terms of orthologs of the mantle transcriptome, we evaluated the assembly with BUSCO against the Metazoa database, with an overall completeness score of 97.4%. In terms of the annotation of the de novo assembly, the final assembly presented a total of 28,720 annotated transcripts. The BLAST2GO functional annotation showed a total of 21,322 annotations on Biological Process (BP), 14,578 annotations on Molecular Functions (MF), and 15,415 annotations on Cellular Component (CC) (Figure S1). Among the annotated biological processes, a large percentage of annotated transcripts were assigned to the cellular process (GO:0009987) and single-organism process (GO:0044699). Among cellular components and molecular function, most of the transcripts were assigned to cell (GO:0005623) and binding (GO:0005488), respectively. Similarly, 9,121 transcripts were mapped to the KEGG database revealing a high number of sequences associated with signal transduction, carbohydrate metabolism, and translation (Figure 2). Other relevant KEGG pathways, highly represented, are amino acid metabolism, the immune system, the endocrine system, and the nervous system. Interestingly, a high percentage of transcripts involved in pathways associated with pigmentation in oysters were identified. For example, 95% of the genes associated with melanogenesis were identified (Figure S2), 85% of the genes associated with tyrosine metabolism were identified (Figure S3), and 91% of the genes associated with calcium signaling pathways were identified (Figure S4).

**Figure 3:**
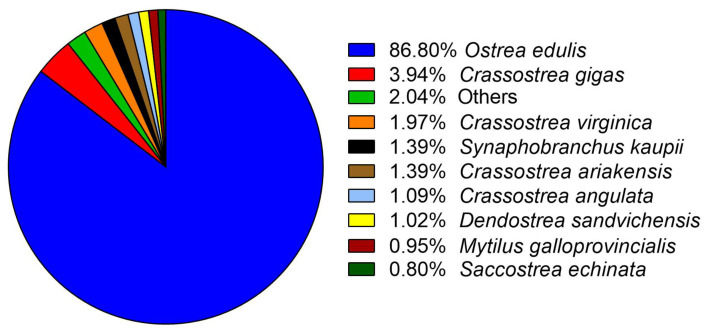
Top-Hits species distribution of BLAST annotation of de novo mantle transcriptome of *Ostrea chilensis*.

The species distribution analysis of transcripts annotation showed that de novo assembly presented high similarity with several oyster species with available genomics sequences such as *Ostrea edulis*, *Crassostrea gigas*, and *Crassostrea virginica* among others (Figure 3).

### 3.2. Assessment of Differentially Expressed Transcripts and Validation of Transcriptomic Data

Differentially expressed transcripts (DETs) were estimated by mapping obtained reads to the mantle assembled transcriptome, resulting in a mapping of 94.3% of total reads. The expression level of each transcript was represented as fold change, with 573 transcripts up-regulated and 173 transcripts down-regulated (Table S4). These DETs were clustered using hierarchy by comparisons between patterns of gene expression (Figure S5).

DETs were analyzed using the DAVID database and categorized as biological process, molecular function, cellular component, and KEGG pathways. DETs were significantly enriched in biological processes, such as translation (GO:0006412), protein folding (GO:0006457), and tricarboxylic acid cycle (GO:0006099) (Table 1). For cellular components, DETs were enriched in cytosolic large ribosomal subunit (GO:0022625), ribosome (GO:0005840), and ribonucleoprotein complex (GO:1990904) (Table 1). For molecular function, DETs were enriched in ATP binding (GO:0005524), RNA binding (GO:0003723), and ATPase activity (GO:0016887) (Table 1).

**Figure 4:**
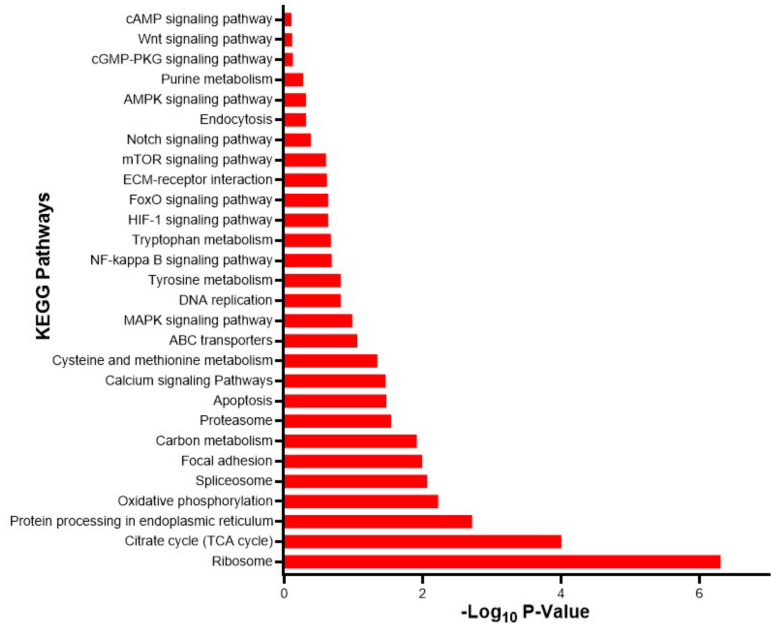
KEGG enrichment of DETs between dark mantle edges (DME) vs. white mantle edges (WME) Chilean oyster mantle transcripts. The graphs show the -log10 of *p*-value enriched of differentially expressed transcripts.

In terms of KEGG enrichment, the most represented pathways include Ribosome, Citrate cycle (TCA cycle), and protein processing in the endoplasmic reticulum. Other interesting pathways include tyrosine metabolism, tryptophan metabolism, cAMP signaling pathway, ABC transporters, Notch signaling pathway, Endocytosis, and Calcium signaling pathway (Figure 4).

**Figure 5:**
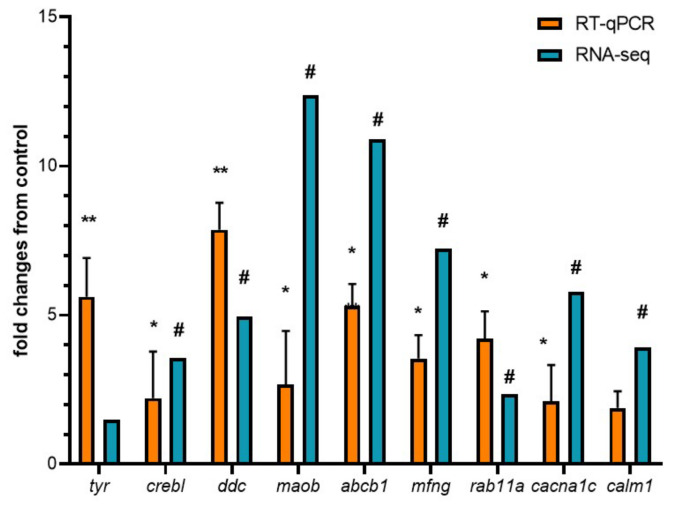
Validation of RNA sequencing by real-time PCR of DETs. The following DETs were selected to validate RNA-seq by real-time PCR: *tyr*, *crebl*, *ddc*, *maob*, *abcb1*, *mfng*, *rab11a*, *cacna1*, *calm1*. For RNA-seq, in blue, “#” indicates a log2 fold change ≥2.0 and FDR <0.05. For RT-qPCR, in orange, relative expression was normalized against *fau* and *actβ*. Fold change from control indicates the relation between dark edge transcript expression and white edge transcript expression. The results are expressed as means and + standard errors (*n* = 3 per group). Differences between DME and WME groups are shown in * *p* < 0.05, and ** *p* < 0.01.

Considering that tyrosine metabolism, tryptophan metabolism, cAMP signaling pathway, ABC transporters, Notch signaling pathway, Endocytosis, and Calcium signaling pathway were overrepresented in KEGG enrichment analysis, we selected nine candidate genes for RT-qPCR validation. We selected Tyrosinase (*tyr*), cAMP Responsive Element Binding protein like (*crebl*), dopa decarboxylase (*ddc*), monoamine oxidase B (*maob*), ATP-binding cassette subfamily B member 1 (*abcb1*), MFNG O-fucosylpeptide 3-beta-N-acetylglucosaminyltransferase (*mfng*), RAS oncogene family (*rab11a*), calcium voltage-gated channel subunit alpha1 C (*cacna1c*), and calmodulin 1 (*calm1*). Our results reveal a high correlation (r = 0.8953; *p* < 0.05) between the expression values of candidate genes using both RNA-seq and qPCR techniques (Figure 5).

## 4. Discussion

In the present article, we report the transcriptome of Chilean flat oyster (*O. chilensis*) mantle using next-generation sequencing (NGS) technology. This is the first report on RNA sequencing, transcriptome assembly, and functional annotation for this species. Our assembled reference transcriptome includes 50,908 transcripts with features similar to those reported in other oyster species. The GC content of *O. chilensis* (37.92%) is slightly lower compared to *Crassostrea gasar* (synonymy *C. tulipa*) (43.68%) [38], *Magallana nippona* (38.57%) [30], and *Crassostrea gigas* (41.7%) [39]. Furthermore, our de novo assembly revealed N50 values and numbers of annotated genes that are similar to or exceed those of pearl oysters, such as *Pinctada maxima* [40], *Pinctada imbricata* [29], and *Pinctada fucata* [41]. These results confirm the high quality of the obtained sequences, as well as the thoroughness of the assembly and functional annotation of the identified genes.

The mantle in oysters plays a crucial role in their anatomy and physiology [42]. It is involved in shell formation, mucous production, pigment distribution, and calcium transportation [43]. The mantle secretes the shell, contains mucous cells that aid in defense against environmental pollutants and pathogens [44], and has sensory cells that detect environmental changes such as predator presence or water quality shifts [45,46]. Additionally, the mantle is responsible for melanin distribution, contributing to the colorful appearance of bivalve shells through specific cellular events that lead to melanin formation and release [47]. Moreover, it is essential for calcium transport and shell maintenance [48]. Interestingly, all these processes related to the metabolism category have a high representation in the transcriptome functional annotation.

A detailed analysis of tissue expression revealed that 746 transcripts are differentially expressed between the dark and white mantle edges of *O. chilensis*. These differentially expressed transcripts are mainly associated with signaling pathways such as Ribosome, Citrate cycle, and protein processing in the Endoplasmic reticulum. Other significant pathways include Tyrosine Metabolism, Tryptophan Metabolism, Endocytosis, Notch Signaling Pathway, Calcium signaling pathway, and ABC transporter. Notably, these pathways are linked to pigmentation regulation in the shell and mantle of bivalves. Research has consistently demonstrated a strong correlation between mantle edge pigmentation and shell color. Brake et al. (2004) reported that pigmentation of the shell and mantle edge in *Crassostrea gigas* is genetically controlled, suggesting that mantle edge pigmentation can be inherited and selected for in breeding programs [14]. Similarly, Han et al. (2022) observed a correlation between mantle edge pigmentation and shell color in the same species. Transmission electron microscopy (TEM) showed melanocytes containing melanosomes below the epidermis of dark mantle tissue, indicating melanin transfer from the mantle to the shell epithelium [49]. A study on *C. gigas* found moderate heritability and a significant genetic correlation between mantle edge and shell pigmentation [50]. Additionally, Zhu et al. (2023) found that the mantle edge of *C. gigas* comprises three folds—outer secretory, middle sensory, and inner muscular—where the outer fold’s melanin contributes to pigmentation [47].

Recent studies have begun to elucidate the molecular bases of oyster mantle pigmentation. Similarly to our study, analyses of the Pacific oyster mantle using RNA-seq identified differences in gene expression related to tyrosine metabolism, melanogenesis, cytochrome P450, endocytosis, and cAMP signaling pathways, which are associated with mantle and shell pigmentation [27,28]. Additionally, a study on the Pacific oyster analyzed the differential expressions of non-coding RNAs involved in biomineralization and shell pigmentation, revealing a complex network in gene expression regulation by modulating RNA stability, and chromatin structure [51]. Notably, LncRNA has been identified as a key regulator of shell biomineralization in *Pinctada fucata* [52], and morphological asymmetry of the shell of species in the genus *Magallana* [53]. The Heme pathway has also shown significant involvement in pigmentation [54], with important changes in gene expression related to the production of red (uroporphyrin and derivatives), yellow (bilirubin), and green (biliverdin and cobalamin forms) pigments in *Pinctada margaritifera var. cumingii* [54].

Among the transcripts validated by RT-qPCR is Tyrosinase (*tyr*). Although no significant changes were observed in RNA-seq analyses, we determined a fold change of 5.62 and 1.5, respectively. Tyrosinase is an enzyme present in plant and animal tissues and catalyzes the production of melanin and other pigments from tyrosine [55]. Its differential activity has been demonstrated in various mantle and shell pigmentation phenotypes in oysters [56]. We also observed significant changes in the expression of transcripts related to tyrosine and tryptophan metabolism. We validated the overexpression of dopa decarboxylase (*ddc*) and monoamine oxidase B (*maob*). MAOB, an enzyme located in the mitochondrial outer membrane, catalyzes the oxidative deamination of biogenic and xenobiotic amines [57]. DDC catalyzes the decarboxylation of L-3,4-dihydroxyphenylalanine (DOPA) to dopamine and L-tryptophan to tryptamine [58]. Tyrosine and tryptophan are aromatic amino acids whose metabolism is linked to the synthesis of various secondary metabolites involved in pigment compounds, hormones, and biological polymers [59]. Although *maob* and *ddc* have not been directly correlated with mantle and shell pigmentation, studies have linked tyrosine and tryptophan metabolism to pigmentation changes [27,28]. Another experimentally validated transcript is the cAMP response element (CRE)-binding protein-like (*crebl*). CREB is a transcription factor that regulates diverse cellular responses, including proliferation, survival, and differentiation [60]. Higher *crebl* expression was found in the mantle of black-shelled Pacific oysters compared to white-shelled ones, mainly associated with melanogenesis [61]. ATP-binding cassette subfamily B member 1 (*abcb1*) encodes a membrane-associated protein involved in the transport of various molecules across membranes, and its overexpression has been linked to pigmentation in *C. gigas* [27]. MFNG O-fucosylpeptide 3-beta-N-acetylglucosaminyltransferase (*mfng*), a member of the glycosyltransferase gene family, acts in the Notch signaling pathway to define boundaries during embryonic development and has been identified as a regulator of the absence of pigmentation in the albino phenotype of *Pinctada margaritifera* [62]. Member RAS oncogene family (*rab11a*) encodes a protein involved in both constitutive and regulated secretory pathways and may be associated with protein transport. This gene has also shown differential expression related to pigmentation in *C. gigas* [27]. Finally, we also analyzed the expression of calcium voltage-gated channel subunit alpha1 C (*cacna1c*) and calmodulin 1 (*calm1*). Both genes have been detected as relevant in calcium signaling and associated with pigmentation in the mantle of *Patinopecten yessoensis* (Yesso scallop) [63].

## 5. Conclusions

The pigmentation of the mantle edge in oysters is a complex trait influenced by both genetic and environmental factors. Understanding the genetic and morphological basis of this trait is essential for breeding programs aimed at selecting specific shell and mantle pigmentation patterns. To the best of our knowledge, this is the first evidence of RNA sequencing, de novo assembly, and functional annotation of the *O. chilensis* transcriptome, as well as the differential expression in the mantle. Gene ontology analysis of transcripts revealed notable differences in the expression profiles between black and white mantle edge pigmentation, allowing the detection of differentially expressed transcripts associated with KEGG pathways like Ribosome, Citrate cycle, Protein processing in the endoplasmic reticulum, Tyrosine metabolism, Tryptophan metabolism, cAMP signaling pathway, ABC transporters, Notch signaling pathway, Endocytosis, and Calcium signaling pathway. The results obtained here will contribute to enriching the genomic resources of *O. chilensis*, improving future molecular and biological studies of this species. In future research, the information obtained will serve as a foundation for identifying novel candidate genes that can facilitate early determination of the white-edge or dark-edge phenotypes in the Chilean flat oyster. This knowledge will contribute to exploring mantle color as a promising trait for selection in upcoming oyster breeding programs.

## Figures and Tables

**Table 1:** Enrichment of DETs in terms of biological process, cellular components, and molecular function in white mantle edge (wme) and dark mantle edge (DME) Chilean oyster groups.

Category	Go Term	Gene Number	*p*-Value
Biological Process	translation	19	1.19 × 10^−10^
	protein folding	14	2.69 × 10^−10^
	tricarboxylic acid cycle	7	4.08 × 10^−06^
	mRNA splicing, via spliceosome	9	2.96 × 10^−04^
	cytoplasmic translation	5	4.68 × 10^−04^
Cellular Component	cytosolic large ribosomal subunit	12	2.79 × 10^−11^
	ribosome	18	2.97 × 10^−10^
	ribonucleoprotein complex	20	4.98 × 10^−10^
	cytosol	33	6.36 × 10^−10^
	cytosolic small ribosomal subunit	8	5.68 × 10^−07^
Molecular Function	ATP binding	68	1.11 × 10^−15^
	RNA binding	41	1.42 × 10^−15^
	ATPase activity	31	3.82 × 10^−15^
	structural constituent of ribosome	19	3.73 × 10^−11^
	unfolded protein binding	13	9.33 × 10^−10^

## Data Availability

The raw read sequences obtained from sequencing were deposited in the Sequence Read Archive (SRA) under BioProject accession number PRJNA1150665 (SRR30335150, SRR30335149). The datasets generated and analyzed during the current study are publicly available.

## Supplementary Materials

The following supporting information can be downloaded at: https://www.mdpi.com/article/10.3390/biology14020145/s1, Figure S1: Gene Ontology categorization (Biological process, Cellular Component, Molecular Function) of transcripts in the mantle transcriptome of *O. chilensis*; Figure S2: Annotated transcripts identified in melanogenesis pathway; Figure S3: Annotated transcripts identified in tyrosine metabolism pathway; Figure S4: Annotated transcripts identified in calcium signaling pathway; Figure S5: Differentially expressed transcripts in dark edges (DE) vs. white edges (WE) Chilean Oyster Mantle. The heatmap plot presents the differentially expressed transcripts between DE and WE. The number indicates the sample library of DE and WE Oyster; Table S1: List of primers used in real-time PCR for RNA-seq validation; Table S2: Summary of transcriptome sequencing for the Chilean Oyster mantle and assembly statistics. bp: base pair; Table S3: Summary of transcriptome assembly statistics. bp: base pair; Table S4: Complete list of DETs for DE and WE comparison.
