# Supplementary figures and images for: Understanding Mantle Edge Pigmentation Through Comprehensive Transcriptomic Profiling of the Chilean Oyster (Ostrea chilensis)
Source: Biology (Basel). 2025 Jan 30;14(2):145. doi: 10.3390/biology14020145 (PMC11852028; doi:10.3390/biology14020145)

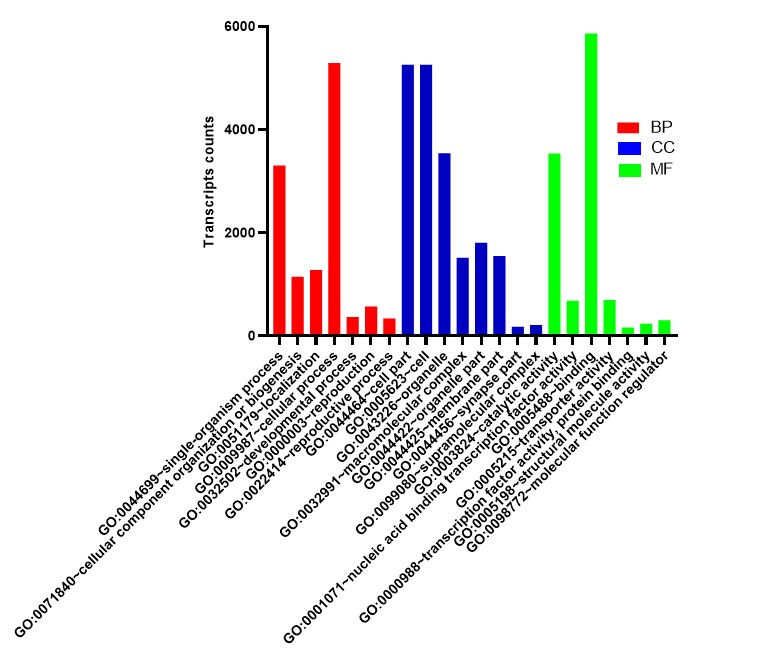

Supplement: Supplementary file 1 [file biology-14-00145-s001.zip › SuplementaryTablesandFigures/FigS1.jpg]

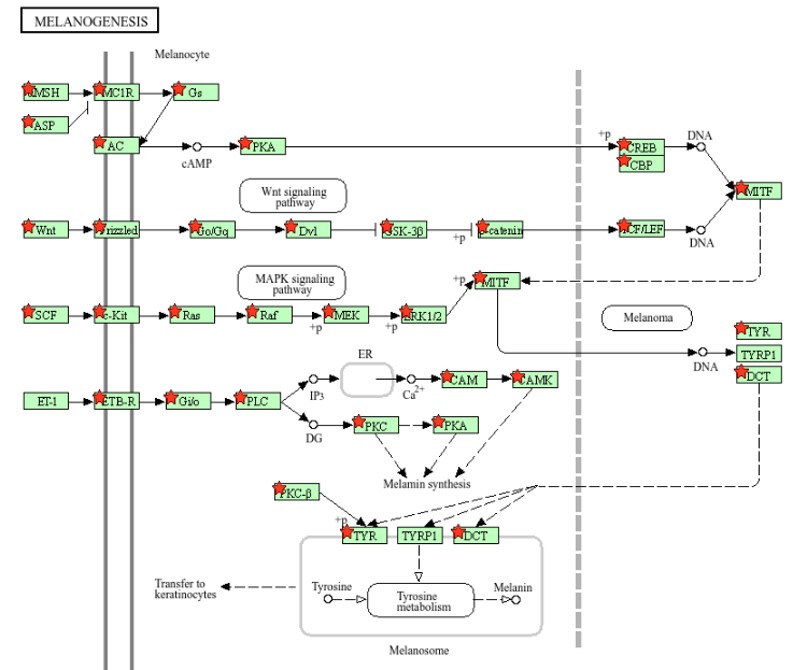

Supplement: Supplementary file 1 [file biology-14-00145-s001.zip › SuplementaryTablesandFigures/FigS2.jpg]

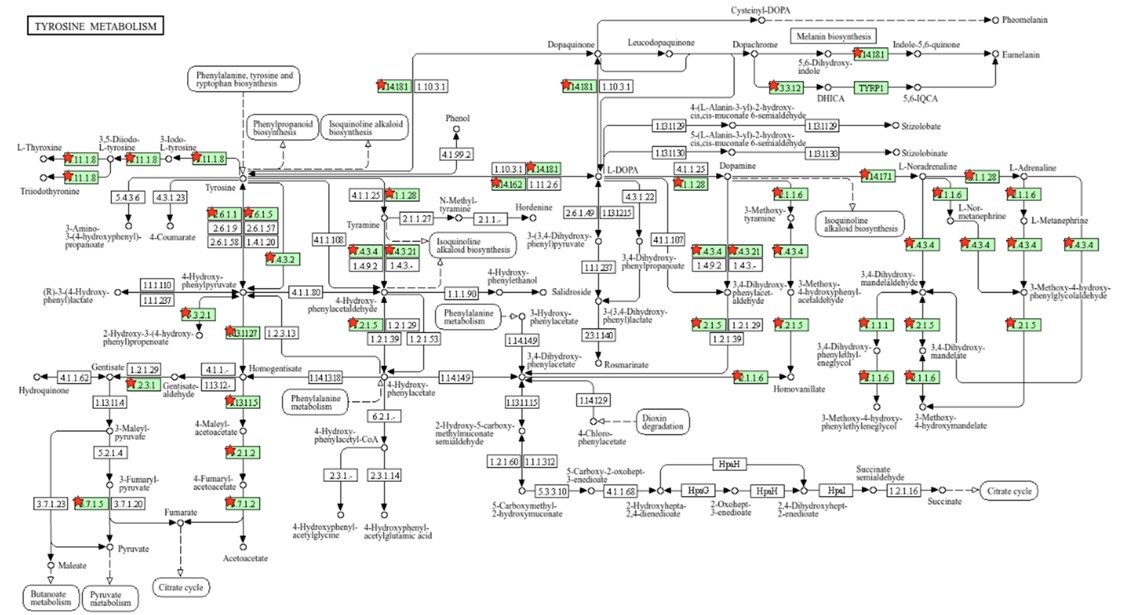

Supplement: Supplementary file 1 [file biology-14-00145-s001.zip › SuplementaryTablesandFigures/FigS3.jpg]

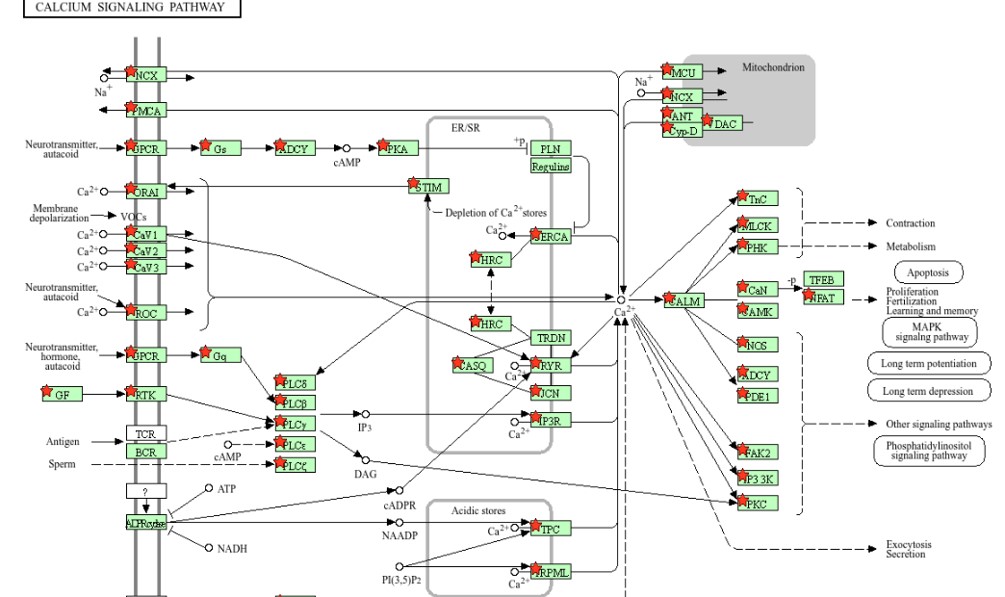

Supplement: Supplementary file 1 [file biology-14-00145-s001.zip › SuplementaryTablesandFigures/FigS4.jpg]

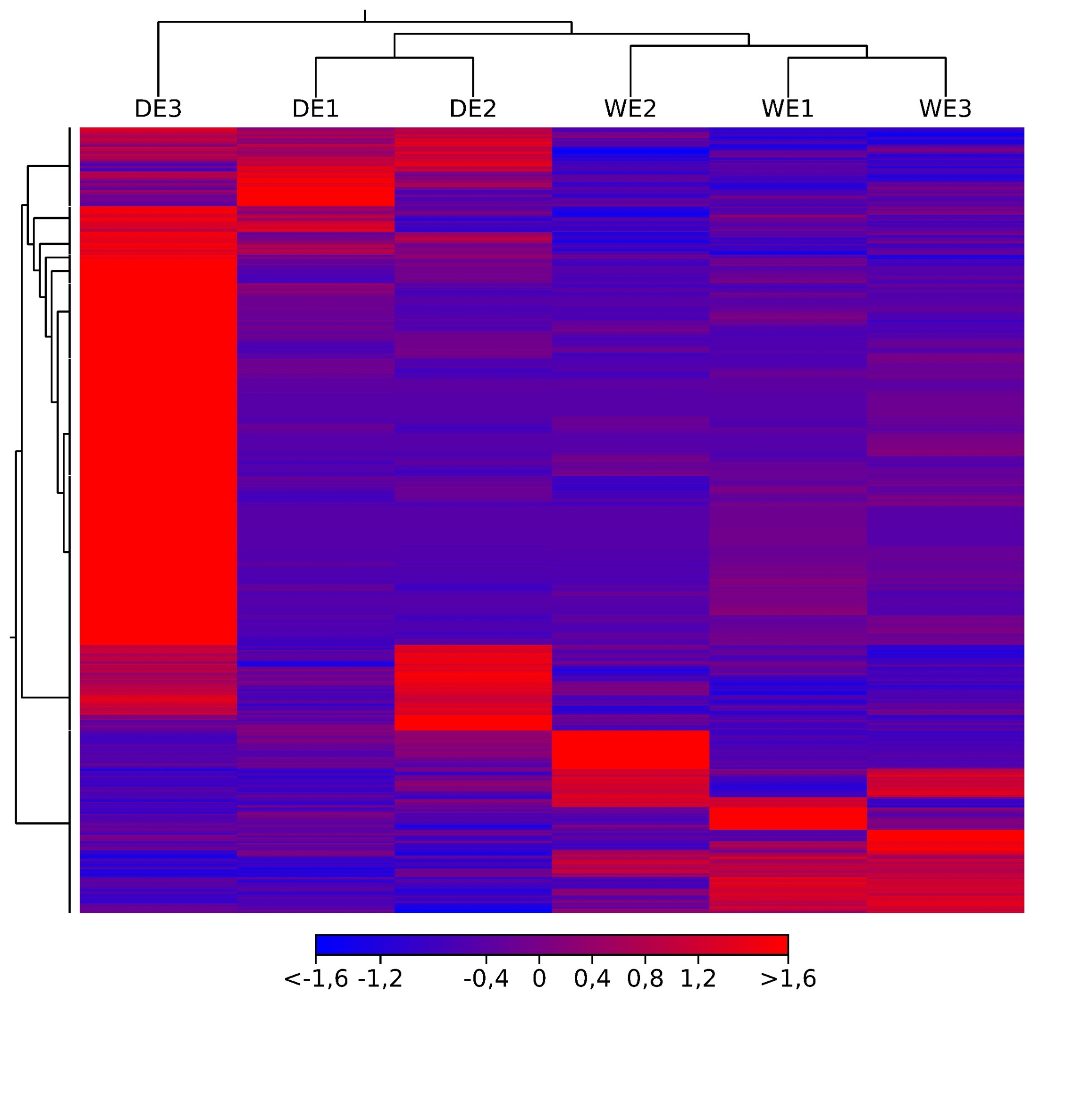

Supplement: Supplementary file 1 [file biology-14-00145-s001.zip › SuplementaryTablesandFigures/FigS5.jpg]
